# Unusual Localization of *Pennella* Sp. in Swordfish (*Xiphias gladius*) Hearts

**DOI:** 10.3390/ani11061757

**Published:** 2021-06-11

**Authors:** Davide Mugetti, Elena Colombino, Vasco Menconi, Fulvio Garibaldi, Walter Mignone, Andrea Gustinelli, Marino Prearo, Franco Guarda, Maria Teresa Capucchio

**Affiliations:** 1 Istituto Zooprofilattico Sperimentale del Piemonte, Liguria e Valle d’Aosta, Via Bologna 148, 10154 Torino, Italy; davide.mugetti@izsto.it (D.M.); waltermignone.wm@gmail.com (W.M.); marino.prearo@izsto.it (M.P.); 2 Department of Veterinary Sciences, University of Turin, Largo P. Braccini 2, 10095 Torino, Italy; elena.colombino@unito.it (E.C.); guarda.franco@gmail.com (F.G.); mariateresa.capucchio@unito.it (M.T.C.); 3 Department of Earth Sciences, Environmental and Life, University of Genova, c.so Europa 26, 16100 Genova, Italy; fulvio.garibaldi@unige.it; 4 Department of Veterinary Medical Sciences, Alma Mater Studiorum University of Bologna, via Tolara di Sopra 50, 40064 Ozzano Emilia, Italy; andrea.gustinelli2@unibo.it

**Keywords:** marine fishes, parasitic diseases, copepods, histological characterization, cardiac inflammation, food safety

## Abstract

**Simple Summary:**

Copepods of the genus *Pennella* are parasites of marine aquatic organisms (e.g., cephalopods, pelagic fish, cetaceans). They can infest fish of economic interest, including tuna and swordfish. The infestation of *Pennella* sp. in fish creates problems of food safety and fish marketing. Generally, these copepods penetrate the host’s muscle, without damaging internal organs. Here, we report on *Pennella* sp. infestation in swordfish heart muscle. The severity of the cardiac lesions we observed suggests that parasites of the genus *Pennella* pose a concern for food safety as well as animal health.

**Abstract:**

The genus *Pennella* comprises hematophagous parasites of marine aquatic species, including cephalopods, marine mammals, and pelagic fish. Nine species have been officially included in the genus *Pennella* plus another six *species inquirendae*. They are most often found in the host’s musculature, without penetrating internal organs. For the present study, 83 hearts from swordfish (*Xiphias gladius*) caught in the Mediterranean Sea were sampled and immediately fixed in formalin for histopathological analysis. In total, 10 (12.05%) hearts were found to be parasitized by copepods of the genus *Pennella*. Macroscopically, there was mild-to-severe fibrinous pericarditis with atrial wall thickening and multiple parasitic nodules. Histologically, the parasitic nodules were surrounded by an inflammatory-necrotizing reaction. Parasitic infestation by *Pennella* spp. is common in pelagic fish and in swordfish, in particular. Here, however, we report atypical cardiac localization. A future area of focus is the evaluation of cardiac *Pennella* spp. infestation by histopathology and genetic identification of the parasites.

## 1. Introduction

*Pennella* (family Pennellidae, order Siphonostomatoida) is a genus of mesoparasitic copepods that infect aquatic animals, including cephalopods, teleosts, and marine mammals [1,2]. *Pennella* species have a heterogenous life cycle: a brief planktonic phase with two naupliar stages, a copepodid stage in which males and pre-metamorphic females develop on the gills of Cephalopoda (e.g., cuttlefish and squids), a chalimus stage, and an adult stage. After mating on the first host, the inseminated females seek a second host (fish or marine mammal) to infest and produce eggs, which are then released into the environment [3]. Nine officially classified species (*P. balaenoptera*, *P. benzi*, *P. diodontis*, *P. exocoeti*, *P. filosa*, *P. hawaiensis*, *P. instructa*, *P. makaira*, *P. sagitta*) and six *species inquirendae* (*P. elegans*, *P. longicauda*, *P. platycephalus*, *P. remorae*, *P. robusta*, *P. selaris*) currently belong to this genus. In addition, other 29 species of *Pennella* not officially recognized are indicated in the literature [4,5]. For these species, the lack of an officially recognized nomenclature is due to single reports, incomplete information, or descriptions similar to species already known but with a different name (e.g., *P. rubra* is synonymous with the well-known *P. filosa*) [6].

Taxonomic descriptions are based on female adult morphology (overall length of the parasite, shape, size, and configuration of the cephalothoracic papillae, segmentation of the first and the second antenna, holdfast horn number, shape and configuration, structure of the abdominal plumes). Nonetheless, it is not always easy to obtain correct identification since adult females are hematophagous and embedded in the musculature of their definitive host [7,8]. The external portion (trunk, genital complex, abdominal plumes) can be easily seen outside the host, while the internal portion (cephalothorax, holdfast horns, neck) is embedded inside the host tissues. This peculiarity makes identification even more complex since the entire parasite needs to be collected for species recognition [5].

The host spectrum, besides morphology, can be an indicator for identifying *Pennella* species. They are highly specific in their host spectrum, having one or two definitive hosts per species [4]. However, *P. balaenoptera* can be found in various marine mammals, including whales, dolphins, porpoises, or pinnipeds [9,10,11,12]. Additionally, *P. filosa* can infest a wide range of marine pelagic fish: dolphinfish (*Coryphaena hippurus*), Indo-Pacific sailfish (*Istiophorus platypterus*), striped marlin (*Kajikia audax*), blue marlin (*Makaira nigricans*), ocean sunfish (*Mola mola*), greater amberjack (*Seriola dumerili*), Atlantic bluefin tuna (*Thunnus thynnus*), albacore (*T. alalunga*), and swordfish (*Xiphias gladius*) [3,13,14,15,16,17].

Although reports are scarce, pelagic species of the Mediterranean may acquire *Pennella* spp. infestation, especially swordfish [18,19]. Adult females of *Pennella* spp. are often found anchored to the fins and fin insertion points of swordfish; occasionally, they may also threaten vital functions by damaging internal organs: the heart, the aorta or other blood vessels, the ovaries, the intestines, and the stomach [18]. The literature on *Pennella* spp. infestation provides little to no information about the histopathological aspect of lesions but rather focuses more on the morphological description [4]. Here, we report on 10 cases of unusual *Pennella* sp. cardiac localization in swordfish.

## 2. Materials and Methods

The study sample was 83 hearts of swordfish fished from the Mediterranean Sea in 2018 for human consumption. After evisceration, the hearts were immediately collected and entirely fixed in 10% neutral buffered formalin (pH 7). A macroscopic external examination was conducted at the Department of Veterinary Sciences, University of Turin. The hearts were sectioned across the median longitudinal plane and representative samples were prepared. The sections were routinely embedded in paraffin wax blocks, sectioned to 5 μm thickness, mounted on glass slides, and stained with haematoxylin and eosin (HE) staining for histological evaluation. Additionally, Weigert–Van Gieson histochemical staining was performed on selected sections. The parasites were macroscopically identified according to published guidelines [4,5].

## 3. Results

**Figure 1:**
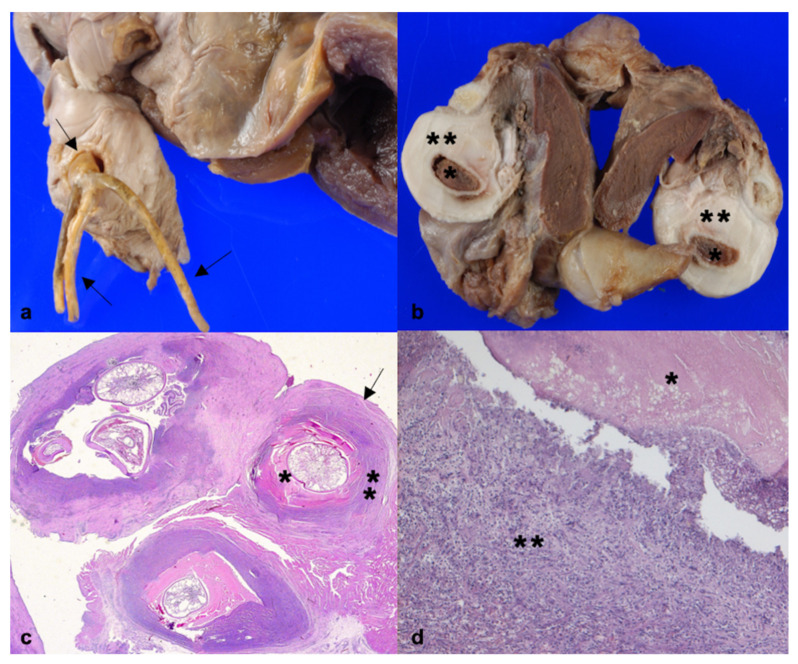
Macroscopic and microscopic features of *Pennella* infestation in the heart of swordfish (*Xiphias gladius*): (**a**) heart, *Pennella* sp. (black arrows) in the atrium; (**b**) heart, cutting surface of parasitic nodules showing a necrotic centre (asterisk) and a thick fibrous capsule (double asterisk); (**c**) heart, multiple parasitic nodules showing parasites surrounded by necrosis (asterisk), inflammation (double asterisk), and a fibrous capsule (black arrow), HE, 50x; (**d**) heart, higher magnification of the necrosis (asterisk) and mixed inflammation (double asterisk) in the myocardium surrounding the parasites (HE, 200x).

At gross examination, a single tubular structure up to 2 cm in length attached to a head with multiple branching “horns” or anchors of up to 1 cm in length compatible with *Pennella* sp. was observed in 10/83 hearts (12.05%) (Figure 1a). Morphological identification to the species level could not be carried out because the parasite was only partially isolated from the heart. Furthermore, biomolecular identification of the parasite was not carried out because no frozen samples were available. On gross examination, mild-to-severe fibrinous pericarditis and serous atrophy of pericardial fat were noted in nine hearts, and no alterations of the pericardium or pericardial fat in one heart. There was marked atrial wall thickening, with single/multiple whitish, firm, irregular nodules from a few millimetres to 2 × 2.5 cm in diameter. The cutting surface of the nodules presented a necrotic centre surrounded by a voluminous fibrous capsule (Figure 1b). No macroscopic alterations of the atrioventricular valves were found. Histologically, there was a moderate, chronic necrotizing-inflammatory reaction, with lymphohistiocytic elements and heterophils infiltrating the myocardium around the parasite (Figure 1c,d). Weigert–Van Gieson staining revealed a thick fibrous capsule surrounding the parasitic nodules.

## 4. Discussion

Infestation with parasites of the genus *Pennella* is not a rare finding in the necropsy of pelagic fish. The genus *Pennella* may pose a serious threat to the swordfish (*Xiphias gladius*), which is one of the commercially most important species on European markets [18]. Previous studies have reported *Pennella* spp. in swordfish caught in the Atlantic Ocean [20,21,22] and in the Mediterranean Sea [23,24]. Here, we report on *Pennella* spp. infestation in the heart of 10/83 swordfish fished from the Mediterranean Sea.

Most previous reports of *Pennella* infestation have described subcutaneous parasitic nodules [13], whereas we noted cardiac localization with moderate chronic necrotizing-inflammatory reaction around the parasitic nodules in the atrial walls. These copepods can occasionally penetrate deeper tissues after digging into the muscle, and sporadic cases have been reported [19]. Parasites, including *Pennella* spp., in commercial fish species have become an emergent food safety problem [7,15,16,17,18]. Our report highlights that *Pennella* sp. can cause serious cardiac damage in swordfish.

Further studies on a wider range of fish species are needed to determine the prevalence of *Pennella* sp. in internal organs and to assess its impact on fish health. In addition, genetic identification should be carried out to determine whether an association exists between *Pennella* species infestation and internal organ lesions.

## Data Availability

Not applicable.
